# Consumption among Cornish Miners

**Published:** 1904-06-18

**Authors:** 


### Consumption Among Cornish Miners.

The inquiry which has recently been made by Mr. Martin, H.M. Inspector of Mines for the South-western District, Dr. Haldane, and Mr. Arthur Thomas into the causes of the excessive mortality among Cornish miners, and the means by which such excessive mortality may be avoided, has by no means proved empty of material result. The inquiry was commenced in October 1902, and it was soon discovered that ankylostomiasis was prevalent among the miners in certain districts in Cornwall, and a report was accordingly made to the Secretary of State and published as a Parliamentary paper in December 1902, in which the prevalence of this disease was stated and some suggestions were offered as to the preventive measures which could be taken. A second report is now published which deals with another illness resulting in excessive mortality among the miners, namely miner's phthisis. In this report are set forth tables by which the death-rate among rock-drillers and other miners may be compared with that obtaining among populations employed in other industries. The figures given reveal several interesting and highly important facts. In the first place they show that while there has been a very great decrease in the death rate among coal miners below the age of 50 during the last half-century, the death-rate among Cornish miners has diminished only at ages below 25. Further, the figures indicate that during recent years the mortality among tin miners between the ages of 25 and 50 has very greatly increased. On analysing the various causes of death it appeared that the excessive mortality among the Cornish miners as compared with that among occupied males generally, and particularly among coal and ironstone miners, was entirely due to lung diseases. Apart from the tendency to pulmonary affections, Cornish mining appears to be an exceptionally healthy occupation, and shows a marked improvement during the last 50 years. Until 1892 the excessive mortality from lung diseases only affected miners of over 40 years of age. Since that time, however, there has been an enormous increase in this kind of mortality among younger men, especially between the ages of 25 and 45, with the result that the total death-rate at all ages from 25 to 55 years is now far greater than at any previous period during the last 50 years. So marked is the excess that in 1900–1902 the annual mortality among miners and mine-labourers between the ages of 35 and 40 in Cornwall was 39·5 per 1,000, or more than three times that of all occupied males of the same age. Further analysis showed that the great increase in the death-rate was chiefly due to deaths from lung disease occurring in men working in connection with rock-drills, and a large proportion of these men had apparently contracted the lung disease while working with machine drills in other countries, and especially in the Transvaal. Among these men the mortality was enormous. With regard to elucidating the nature of miner's phthisis, the authors of the report state that they were unable to accomplish much owing to the existing prejudice in Cornwall against post-mortem examinations, which rendered it impossible for them to obtain permission for a single autopsy on any of the numerous miners who died during the inquiry. They were further considerably handicapped by the absence of any local hospital in which cases of miner's phthisis could be carefully observed. However, it appears that miner's phthisis is tuberculosis occurring in lungs injured by the inhalation of hard stone-dust and occurring especially among men engaged in working rock-drills. The essential measure of prophylaxis is therefore to prevent the inspiration of the rock-dust, and a completely effective remedy for this, the report states, is afforded by a jet or spray of water passing into the holes, only a small jet of water being needed to entirely prevent the issue of dust capable of floating in the air. By a jet of water, also, the air may be readily cleared of dust raised by blasting; and it is suggested for the same purpose that the ore should be damped before it is moved.

It may be hoped that no time will be lost in giving practical shape to the suggestions embodied in the report, and that the Cornish mine-owners, by supplying the necessary apparatus and by ensuring that their workmen are properly instructed in personal hygiene, will at once endeavour to fulfil the obligations imposed upon them of protecting the lives of the workers upon whom their own prosperity depends. Neglect of the simple remedies suggested would be equivalent to wholesale manslaughter.

